# Correction: Targeting a highly repetitive genomic sequence for sensitive and specific molecular detection of the filarial parasite *Mansonella perstans* from human blood and mosquitoes

**DOI:** 10.1371/journal.pntd.0011096

**Published:** 2023-01-26

**Authors:** The PLOS Neglected Tropical Diseases Staff

There is an error in the affiliation of Editor Pablo Maravilla. The correct affiliation is Hospital General Dr. Manuel Gea González, MEXICO.

## References

[1] Pilotte N, Thomas T, Zulch MF, Sirois AR, Minetti C, Reimer LJ, et al. (2022) Targeting a highly repetitive genomic sequence for sensitive and specific molecular detection of the filarial parasite *Mansonella perstans* from human blood and mosquitoes. PLoS Negl Trop Dis 16(12): e0010615. 10.1371/journal.pntd.0010615 36580452 PMC9833530

